# Peer review of "Increased Density of Growth Differentiation Factor-15+ Immunoreactive M1/M2 Macrophages in Prostate Cancer of Different Gleason Scores Compared with Benign Prostate Hyperplasia"

_cancers, 2022, doi:10.3390/cancers14194591_

Round 1
Reviewer 1 Report
The authors present a well-designed and well-presented study on clinically obtained specimens suggesting that monitoring GDF-15 in prostate cancer, or in urine or serum, might represent a yet unrecognized means aiding physicians in detecting and classifying prostate cancer, in addition to the classically used PSA test (of note: naturally, a few follow up studies would be needed). This study is therefore of high significance and therefore interesting to the field.
The study also reveals interesting aspects regarding the tumor environment and innervation of PCa.
Methods are explained clearly and ethical approval for this study has been granted.
The results are presented in a logical manner, with all figures being clear, analyzed properly, which also extends to the proper statistical analyses.
The main parts of the manuscript are written nicely with very few errors (check line 141, 268,274, 345, 377, 412) and introduce, present, and discuss the results in an appropriate manner.
I only have one question/suggestion: even though the authors use antibodies from rather trusted sources, I am wondering if the applied antibodies have been tested in immunoblotting on pertinent lysates. If yes, this should be mentioned in the M&M sections and such blots should be provided in the supplemental figures.
Other than that I have no further concerns.
Author Response
Review1 Report Form
Comments and Suggestions for Authors
The authors present a well-designed and well-presented study on clinically obtained specimens suggesting that monitoring GDF-15 in prostate cancer, or in urine or serum, might represent a yet unrecognized means aiding physicians in detecting and classifying prostate cancer, in addition to the classically used PSA test (of note: naturally, a few follow up studies would be needed). This study is therefore of high significance and therefore interesting to the field.
The study also reveals interesting aspects regarding the tumor environment and innervation of PCa.
Methods are explained clearly and ethical approval for this study has been granted.
The results are presented in a logical manner, with all figures being clear, analyzed properly, which also extends to the proper statistical analyses.
The main parts of the manuscript are written nicely with very few errors (check line 141, 268,274, 345, 377, 412) and introduce, present, and discuss the results in an appropriate manner.
I only have one question/suggestion: even though the authors use antibodies from rather trusted sources, I am wondering if the applied antibodies have been tested in immunoblotting on pertinent lysates. If yes, this should be mentioned in the M&M sections and such blots should be provided in the supplemental figures.
Other than that I have no further concerns.
Submission Date
02 August 2022
Date of this review
12 Aug 2022 04:50:38
ANSWERS
REV1: The main parts of the manuscript are written nicely with very few errors (check line 141, 268,274, 345, 377, 412) and introduce, present, and discuss the results in an appropriate manner.
RE: According to the reviewer’s criticism we have – once again - reviewed the manuscript to our best by including a native English-speaker of our group; in this context, the following errors have been accordingly corrected, as also highlighted in the revised manuscript in green colour:
“density significantly correlated positively” (s. page 1, line 40)
“Gleason” (s. page 2, line 48)
“deliver the histological diagnosis” (s. page 3, line 60)
“reported to be positively associated” (s. page 3, lines 68-69)
“has most recently been proposed” (s. page 3, line 100)
“injuries or patients treated” (s. page 4, line 122)
“All procedures in this study” (s. page 4, line 129)
“implicit consent to make use” (s. page 4, line 132)
“diagnosis of BPH were carried” (s. page 4, line 149)
“neuropeptides with a direct action” (s. page 12, line 329)
“However, epithelial and” (s. page 13, line 336)
“Here, for the first time, we show” (s. page 13, lines 353-354)
“Here, for the first time, we show” (s. page 13, line 368)
"data showing the localization” (s. page 13, line 374)
“an association between low M2-MΦ” (s. page 13, line 381)
“IR was not localized” (s. page 14, line 389)
“becomes evident in the following” (s. page 14, line 390)
“detection of PCa, or” (s. page 14, line 412)
“used as an additional” (s. page 14, line 415)
“and the presence PD-L1” (s. page 14, line 417)
“excrescences remains to be shown” (s. page 14, line 418)
REV1: I only have one question/suggestion: even though the authors use antibodies from rather trusted sources, I am wondering if the applied antibodies have been tested in immunoblotting on pertinent lysates. If yes, this should be mentioned in the M&M sections, and such blots should be provided in the supplemental figures.
RE: The antibodies used in this study have been already validated, previously been published (see list) and have been used routinely in our histology lab. Moreover, validating these antibodies e.g. by using WB was impossible, because formalin-fixed and paraffin-embedded biopsies of PCa and BPH were delivered by the Clinic of Urology /Dept. of Pathology (Marburg University). Thus, it was impossible to generate homogenates of the biopsies before fixation in paraffin. In addition, producing homogenates of the samples, which have already been included in paraffin to perform WB, does not provide any more information than the direct use of antibodies in histological sections, by using antibodies that have been used for immunohistochemical investigations and published for several times in the past by other scientists (see list below).
Mouse anti-human CD4, MAB379, R&D Systems, Minneapolis, USA.
Chimote AA, Hajdu P, Sfyris AM, Gleich BN, Wise-Draper T, Casper KA, Conforti L. Kv1.3 Channels Mark Functionally Competent CD8+ Tumor-Infiltrating Lymphocytes in Head and Neck Cancer. Cancer Res. 2017 Jan 1;77(1):53-61. doi: 10.1158/0008-5472.CAN-16-2372. Epub 2016 Nov 4. PMID: 27815390; PMCID: PMC5215046.
Mouse anti-human CD8, IHCR2114-6, EMD Millipore, Billerica, USA
Sznurkowski JJ, Żawrocki A, Krawczyńska N, Bieńkowski M, Wasąg B, Biernat W. Impact of Activation of EGFL7 within Microenvironment of High Grade Ovarian Serous Carcinoma on Infiltration of CD4+ and CD8+ Lymphocytes. Medicina (Kaunas). 2022 Apr 24;58(5):588. doi: 10.3390/medicina58050588. PMID: 35630004; PMCID: PMC9144271.
Mouse anti-human CD19, MCA2454T, Bio-Rad, Kidlington, UK.
Streeck H, Kwon DS, Pyo A, Flanders M, Chevalier MF, Law K, Jülg B, Trocha K, Jolin JS, Anahtar MN, Lian J, Toth I, Brumme Z, Chang JJ, Caron T, Rodig SJ, Milner DA Jr, Piechoka-Trocha A, Kaufmann DE, Walker BD, Altfeld M. Epithelial adhesion molecules can inhibit HIV-1-specific CD8⁺ T-cell functions. Blood. 2011 May 12;117(19):5112-22. doi: 10.1182/blood-2010-12-321588. Epub 2011 Mar 14. PMID: 21403126; PMCID: PMC3109536.
Mouse anti-human CD68, M0876, Dako, Glostrup, Dänemark
Falini B, Flenghi L, Pileri S, Gambacorta M, Bigerna B, Durkop H, Eitelbach F, Thiele J, Pacini R, Cavaliere A, et al. PG-M1: a new monoclonal antibody directed against a fixative-resistant epitope on the macrophage-restricted form of the CD68 molecule. Am J Pathol. 1993 May;142(5):1359-72. PMID: 7684194; PMCID: PMC1886928.
Mouse anti-human CD163, ab74604, Abcam, Cambridge, UK
Tjomsland V, Niklasson L, Sandström P, Borch K, Druid H, Bratthäll C, Messmer D, Larsson M, Spångeus A. The desmoplastic stroma plays an essential role in the accumulation and modulation of infiltrated immune cells in pancreatic adenocarcinoma. Clin Dev Immunol. 2011;2011:212810. doi: 10.1155/2011/212810. Epub 2011 Dec 6. PMID: 22190968; PMCID: PMC3235447.
Rat anti-human GDF-15, ab189358, Abcam, Cambridge, UK
Wang T, Liu J, McDonald C, Lupino K, Zhai X, Wilkins BJ, Hakonarson H, Pei L. GDF15 is a heart-derived hormone that regulates body growth. EMBO Mol Med. 2017 Aug;9(8):1150-1164. doi: 10.15252/emmm.201707604. PMID: 28572090; PMCID: PMC5538424.
Rabbit anti- human PD-L1 (CD274), 13684, Cell Signaling Technology, Inc. Danvers, USA
Mahmoud AM, Frank I, Orme JJ, Lavoie RR, Thapa P, Costello BA, Cheville JC, Gupta S, Dong H, Lucien F. Evaluation of PD-L1 and B7-H3 expression as a predictor of response to adjuvant chemotherapy in bladder cancer. BMC Urol. 2022 Jun 24;22(1):90. doi: 10.1186/s12894-022-01044-1. PMID: 35751046; PMCID: PMC9233321.
Rabbit anti-human PGP9.5 Ubiquitin C-Terminal Hydrolase L1 (UCHL1), CL95101 Cedarlane, Burlington, USA
Anlauf M, Schäfer MK, Eiden L, Weihe E. Chemical coding of the human gastrointestinal nervous system: cholinergic, VIPergic, and catecholaminergic phenotypes. J Comp Neurol. 2003 Apr 21;459(1):90-111. doi: 10.1002/cne.10599.

Reviewer 2 Report
Please find the attached file.

Author Response
Review2 Report Form
RE: First of all, we wish to thank the reviewer for constructive criticism.
- Simple summary:
- The study aim was not totally clear: The author started with the statement
“The main challenge is to discover biomarkers for malignancy to guide the
physician towards optimized diagnosis and therapy.”. This could be
interpreted as the purpose of this study is to differentiate high GS (6-9)
patients by using proposed biomarker (GDF-15+).
Yet the following
statement “Here we show that the density of GDF-15+ cells mainly
identified as interstitial macrophages was higher in GS6-9 than in BPH.”
Can only be interpreted as GDF-15+ biomarker is intended to differentiate
high GS patients vs BPH patients. If this is the case, please clearly convey
how GDF-15+ could be used as a potential biomarker and what was the
challenge.
RE: Changed as suggested,
from
“Here we show that the density of GDF-15+ cells mainly identified as 24 interstitial macrophages was higher in GS6-9 than in BPH.”
To
“Here we show that the density of GDF-15+ cells, mainly identified as interstitial macrophages (MΦ), was higher in GS6-9 than in BPH, and, thus, GDF-15 is intended to differentiate patients with high GS vs BPH, as well as GS6 vs GS7 (or even with higher malignancy)(s. lines 24-26)”
- b. Row 25-26, “Some GDF-15+ macrophages showed transepithelial
migration into glandular lumen.” Please clarify what is the clinical
relevance? Urine/Semen detection?
RE: Changed as suggested,
from
“Some GDF-15+ macrophages showed transepithelial migration into glandular lumen.”
To
“Some GDF-15+ MΦ showed a transepithelial migration into the glandular lumen and , thus, might be used to be measured in urine/semen. (s. lines 27-28).”
- The sentence in row 27-28 indicated the use of GDF-15 as a tool to
differentiate BPH patients vs PCa patients, which does not resolve the
challenge of discover biomarker for malignancy. (Same as described
above)
RE: Changed as suggested (see also comment above),
from
“Thus, GDF-15 is proposed as a new tool to diagnose and stratify the malignancy of PCa and as a potential target for anti-tumor therapy.”
To
“Taken together, GDF-15 is proposed as a novel tool to diagnose PCa vs BPH or malignancy (GS6 vs higher GS) and as a potential target for anti-tumor therapy (s. line 28-29).”
- Abstract:
- a. Row 29, add a comma after PCa
RE: Changed as suggested.
“PCa, (s. line 32)”
- Row 42, please make change “the degree of GDF-15 increases in
M1/M2Φ” into “the degree of GDF-15 increases in extratumoral M1/M2Φ”
RE: Changed as suggested,
from
“The degree of GDF-15 increases in M1/M2Φ is proposed to be useful to stratify progredient malignancy of PCa. GDF-15 is a potential target for anti-tumor therapy.”
To
“The degree of extra-/intra-tumoral GDF-15 increases in M1/M2Φ is proposed to be useful to stratify progredient malignancy of PCa. GDF-15 is a potential target for anti-tumor therapy. (s. line 45-47).”
- Intro:
- Statement from row 64-65, re-word the sentence: positively associated or
negatively associated; good / bad clinical outcome
RE: Changed as suggested,
from
“However, infiltration of immune cells in PCa has been reported to be strongly associated with clinical outcomes [6]”
To
“However, infiltration of immune cells in PCa has been reported to be positively associated with clinical outcomes [6]” (s. page 3, lines 68-69)”
- Tissue Specimens and (Immuno)histochemistry (IHC)
- Row 135-136, please described the stage of tumor of specimens’ origin
(GS6-9) to differentiate samples from spreading vs non-spreading tumor.
This could result in different infiltrated immune cells population in ET vs IT.
RE: Changed as suggested,
from
Additionally, the use and examination of the PCa and BPH biopsies were approved by the Ethics committee of the medical faculty of the Philipps University of Marburg (AZ: Study 95/15).
To
Additionally, the use and examination of the PCa (GS6-GS9) and BPH biopsies were approved by the Ethics committee of the medical faculty of the Philipps University of Marburg (AZ: Study 95/15). (s. page 4, lines 136-138).
The histopathological classification of the PCa according to Gleason and the differential diagnosis of BPH were carried out by specialists of the institute for pathology at the UKGM Marburg, who also defined intratumoral (IT) and extratumoral (ET) regions of every single biopsy.” See also page 4, lines 151-154.
- The changed PGP9.5+-innervation matrix in PCa of different GS as compared to
BPH
- PGP 9.5+ density results suggested significance between BPH vs GS9
and GS6 vs GS9, yet no correlation analysis (Correlation co-efficiency and
p-value) was conducted to support statement in Row 328-330 “almost
linearly increasing”. In addition, “almost linearly increasing” is a vague
statement, please re-word.
RE: Changed as suggested
from
“We found in all PCa and BPH samples, an extensive network of PGP9.5+ nerve fibers and cells, with lowest PGP9.5+ areas in BPH, almost linearly increasing in PCa from GS6 to GS9.”
to
“We found in all PCa and BPH samples, an extensive network of PGP9.5+ nerve fibers and cells, with the lowest PGP9.5+ areas in BPH, increasing in PCa from GS6 to GS9.” (s. page 13, lines 337-339)
- Other than looking for the result conclusion in discussion and conclusion
section, please add a brief conclusion in row (188).
RE: Sorry, but we do not really understand what the reviewer is suggesting since line 188 is in the “results” section and neither in the “discussion” nor in the “conclusion” sections.
However, we wish to mention that in the original version of the manuscript, we have already written in the “results” section
Line 188: Immunoreactivity (IR) for PGP9.5 was found in nerve fibers and NE cells in PCa of all GS.
Moreover, in the “discussion” section, we have already mentioned:
“PGP9.5 is not only found in nerves but also prostatic NE cells [34]. If released from NE cells in prostatic cancer PGP9.5 may exert paracrine effects to promote the proliferation of neighbouring cells [34].” (s. page 13, lines 347-349)
Thus, we would appreciate receiving detailed information about the criticism. Does the reviewer really suggest to include a brief conclusion in the “results” section? If so: Where?
- Increased density of GDF-15+ cells in PCa of different GS as compared to BPH
- What is the significance level of GS8
RE: When comparing the density of GDF-15+ cells in PCa GS8 with BPH, we found a P-value of 0.230 (not significant). Please find the statistical analyses below.
Data source: Data 1 in Notebook1-GDF15-MS-GAB
Normality Test (Shapiro-Wilk) Passed (P = 0,243)
Equal Variance Test: Failed (P < 0,050)
Test execution ended by user request, Rank Sum Test begun
Mann-Whitney Rank Sum Test Freitag, August 26, 2022, 12:00:42
Data source: Data 1 in Notebook1-GDF15-MS-GAB
Group N Missing Median 25% 75%
GDF15-G8-ET+IT 8 1 4,074 0,000 6,030
GDF15-BPH 4 0 0,0111 0,000 0,0559
Mann-Whitney U Statistic= 7,000
T = 17,000 n(small)= 4 n(big)= 7 P(est.)= 0,209 P(exact)= 0,230
The difference in the median values between the two groups is not great enough to exclude the possibility that the difference is due to random sampling variability; there is not a statistically significant difference (P = 0,230)
- Increased density of CD68+ and CD163+ macrophages in PCa of different GS as
compared to 209 BPH
- Please comment on the population density of CD68 reside in IT and ET. If
the majority population are localized in ET, this will strengthen the
correlation between CD68 ET and GDF-15 ET for potential use as
biomarker.
RE: According to the reviewer’s suggestion, we have now added to the results/discussion sections of the revised MS:
- page 7, lines 229-232
“However, densities of ET or IT CD68+ MΦ did not significantly correlate with the density of IT GDF-15+ cells (Figure 3C,D). The densities of CD68+, CD163+, and GDF15+ cells in IT regions compared with those in ET areas were insignificantly different (data not shown).”
page 13, lines 370-373:
“The densities of CD68+ and GDF15+ cells in IT regions were insignificantly higher than in ET regions. However, the positive correlation between the density of CD68+ MΦ and GDF-15+ cells in ET areas and the presence in the luminal excrescences might be a sign to consider the use of GDF-15 as a potential biomarker.”
- Same with CD163 ET vs IT population density
RE: changed/optimized: See comment above
- Figure 3D & 4D, P value and r value is switched.
RE: Thank you for your suggestion. According to the reviewer’s criticism, we have corrected figures 3 and 4. (s. pages 8 and 9)
- Preferential localization of GDF-15 in transepithelial migrating CD68+ or CD163+
MΦ and in PGP9.5+ epithelial cells in PCa
- What is the significance level of co-localization?
RE: Our interesting observations have to date only been morphologically performed. Colocalization was not quantified, and significance was not calculated. Because tissue material (biopsies) is limited at the moment, this should be done in future studies.
- Relationship of GDF-15, PD-L1, CD68 and CD163 in luminal excrescences in
PCa 275 compared to BPH
- Does (GS7-9) following the same pattern?
RE: We found luminal excrescences in all stages of PCa. However, with differences in the colocalization pattern of GDF-15 and PDL1. Not all excrescences were GDF-15+ and PDL1+, and some were only PDL1 or GDF15+ or negative for both markers. However, in BPH, no excrescences with GDF-15+ and PDL1+ cells were found at all. From GS6-up to GS9, GDF15+ and PD-L1+ excrescences were found but markedly increased in GS9.
According to the reviewer’s suggestion, we have now added to the discussion sections of the revised MS (s. page 14 lines 418-423):
“It is noteworthy to mention that we found luminal excrescences in all stages of PCa. However, with differences in the colocalization pattern of GDF-15 and PDL1. Not all excrescences were GDF-15+ and PDL1+, and some were only PDL1+ or GDF15+ or negative for both markers. However, in BPH, GDF-15+ and PDL1+ cells were never found in excrescences. From GS6 -up to GS9, GDF15+ and PD-L1+ excrescences were found and markedly increased in GS9.”
10.Decreased density of CD4+ lymphocytes in PCa compared with BPH and close
contact of 289 CD19+ B-lymphocytes with nerves in PCa but not in BPH
- Why is this not co-stained with GDF-15
RE: Lymphocytes can be easily identified by their morphology, and we have never seen a GDF-15+ immunoreaction in lymphocytes in any biopsy.
11.Discussion
- Please discuss the practical use of GDF-15 as biomarker
RE: According to the reviewer's criticism, we have added to the “discussion” section:
“As PD-L1 is a known marker of tumor progression [64,65], our findings of the colocalization of PDL1 with GDF-15 in PCa but not in BPH is a totally novel discovery that could be used in future, prospective studies as biomarkers of malignancy in tissue biopsies or cells/RNA in urine or seminal plasma [66,67]. (s. page 14, lines 423-427).
And the references (s page 18 lines 620-629)
- He, J.,Yi, M, Tan, L, Huang, J., Huang, L. The immune checkpoint regulator PD-L1 expression are associated with clinical progression in prostate cancer. World J. Surg. Oncol. 2021, 19, 215. doi: 10.1186/s12957-021-02325-z.
- Ye, L., Zhu, Z., Chen, X., Zhang, H., Huang, J., Gu, S., Zhao, X. The Importance of Exosomal PD-L1 in Cancer Progression and Its Potential as a Therapeutic Target. Cells. 2021, 10, 3247. doi: 10.3390/cells10113247.
- Rubio-Briones, J, Borque-Fernando, A., Esteban, L.M., Mascarós, J.M., Ramírez-Backhaus, M., Casanova, J., Collado, A., Mir, C., Gómez-Ferrer, A., Wong, A.; et al. Validation of a 2-gene mRNA urine test for the detection of ≥GG2 prostate cancer in an opportunistic screening population. Prostate. 2020, 80(6):500-507. doi: 10.1002/pros.23964.
- Lorente, G., Ntostis, P., Maitland, N., Mengual, L., Musquera, M., Muneer, A., Oliva, R., Iles, D., Miller, D. Semen sampling as a simple, noninvasive surrogate for prostate health screening. Syst Biol Reprod Med. 2021, 67, 354-365. doi: 10.1080/19396368.2021
12.Conclusion
- Row 408-409, the summary statement is not accurate. (i.e. CD68+ does
not necessarily increase according to the malignancy level)
RE: According to the reviewer’s criticism we have changed the text
From
“In summary, the current study indicates that density of innervation, CD68+ (M1), CD163+(M2) MΦ and GDF15+ cells increases in PCa according to the degree of malignancy.”
To
“In summary, the current study indicates that density of innervation, CD68+ (M1), CD163+(M2) MΦ and GDF15+ cells partly increases in PCa according to the degree of malignancy.” (s. page 14, lines 429-431)
- The author stated that GGF-15 can be found in seminal plasma and/or
urine, yet did not provide evidence to support the level of difference
between BPH vs PCa. Therefore, the conclusion of using it as a potential
biomarker cannot be drawn.
RE: We do not affirm that this is the case, but rather that it would be possible to use this characteristic as a biomarker of PCa malignancy. Undoubtedly the hypothesis has to be confirmed in future, prospective studies by investigation of urine samples and seminal plasma. Thus, we just hypothesize or propose as follows:
“The occurrence of GDF-15+ cells in epithelial excrescences of PCa suggests that GDF-15 can be found in seminal plasma/and or urine. Thus, GDF-15 in seminal plasma/and/or urine could be utilized as a non-invasive biomarker of PCa as compared to BPH.” (s. page 14, lines 432-435)
Moreover, we have added to the “conclusions” section:
“Nevertheless, future/prospective clinical studies are necessary to test our hypotheses by using an increased number of patients.” (s. page 14, lines 437-439).
- The author did not provide evidence suggesting how co-presence of PDL1 & GDF-15 can be used to define tumor grade progression.
RE: In this regard, there are several recent publications on the importance and relationship between PD-L1 and cancer progression, as well as PD-L1 expression and prostate cancer progression (64, 65). PD-L1 as a marker of tumorigenicity has recently been published (64, 65), but the colocalization with GDF-15 is also a totally novel discovery, indicating that GDF-15 could be used as a marker of malignancy in tissue biopsies or cells/RNA in urine or seminal plasma (66, 67). According to the reviewer's comment, we have set out our position in the following paragraph in the “discussion” section:
“As PD-L1 is a known marker of tumor progression [64,65], our findings of the colocalization of PDL1 with GDF-15 in PCa but not in BPH is a totally novel discovery that could be used in future, prospective studies as biomarkers of malignancy in tissue biopsies or cells/RNA in urine or seminal plasma [66,67].” (s. page 14, lines 423-427).
We have also added the references (s page 18 lines 620-629) to the revised manuscript:
- He, J.,Yi, M, Tan, L, Huang, J., Huang, L. The immune checkpoint regulator PD-L1 expression are associated with clinical progression in prostate cancer. World J. Surg. Oncol. 2021, 19, 215. doi: 10.1186/s12957-021-02325-z.
- Ye, L., Zhu, Z., Chen, X., Zhang, H., Huang, J., Gu, S., Zhao, X. The Importance of Exosomal PD-L1 in Cancer Progression and Its Potential as a Therapeutic Target. Cells. 2021, 10, 3247. doi: 10.3390/cells10113247.
- Rubio-Briones, J, Borque-Fernando, A., Esteban, L.M., Mascarós, J.M., Ramírez-Backhaus, M., Casanova, J., Collado, A., Mir, C., Gómez-Ferrer, A., Wong, A.; et al. Validation of a 2-gene mRNA urine test for the detection of ≥GG2 prostate cancer in an opportunistic screening population. Prostate. 2020, 80(6):500-507. doi: 10.1002/pros.23964.
- Lorente, G., Ntostis, P., Maitland, N., Mengual, L., Musquera, M., Muneer, A., Oliva, R., Iles, D., Miller, D. Semen sampling as a simple, noninvasive surrogate for prostate health screening. Syst Biol Reprod Med. 2021, 67, 354-365. doi: 10.1080/19396368.2021
