# Supplementary material for: Increased Density of Growth Differentiation Factor-15+ Immunoreactive M1/M2 Macrophages in Prostate Cancer of Different Gleason Scores Compared with Benign Prostate Hyperplasia
Source: Cancers (Basel). 2022 Sep 22;14(19):4591. doi: 10.3390/cancers14194591 (PMC9578283; doi:10.3390/cancers14194591)
Supplement: Supplementary file 1 [file cancers-14-04591-s001.zip › cancers-1873035-supplementary.pdf]

cancers

MDPI

Supplementary Materials:

Name	Cat. number	Company
Primary antibodies		
Mouse anti-human CD4	MAB379	R&D Systems, Minneapolis, USA.
Mouse anti-human CD8	IHCR2114-6	EMD Millipore, Billerica, USA
Mouse anti-human CD19	MCA2454T	Bio-Rad, Kidlington, UK.
Mouse anti-human CD68	M0876	Dako, Glostrup, Dänemark
Mouse anti-human CD163	ab74604	Abcam, Cambridge, UK
Rat anti-human GDF-15	ab189358	Abcam, Cambridge, UK
Rabbit anti-human PD-L1 (CD274)	13684	Cell Signaling Technology, Inc. Danvers, USA
Rabbit anti-human PGP9.5 Ubiquitin C-Terminal Hydrolase L1 (UCHL1)	CL95101	Cedarlane, Burlington, USA
Secondary antibodies		
Rabbit anti-mouse-AP	ab6729/ab7077	Abcam, Cambridge, UK
Donkey anti-rabbit-biotin	711-005-152	Jackson I.R.E. Ltd. Cambridge, UK
Goat anti-rabbit-HRP	ZRH 1158	Linaris GmbH, Mannheim, Germany
Goat anti-rat-AP	112-055-003	Dianova, Hamburg, Germany
Donkey anti-mouse-biotin	715-165-151	Dianova, Hamburg, Germany
Donkey anti-rat-biotin	712-165-153	Dianova, Hamburg, Germany
**Immunofluorescence double staining**		
Mouse anti-human CD68	M0876	Dako, Glostrup, Dänemark
Mouse anti-human CD163	ab74604	Abcam, Cambridge, UK
Rat anti-human GDF-15	ab189358	Abcam, Cambridge, UK
Streptavidin, Alexa Fluor™ 488	S 11223	Invitrogen, Karlsruhe, Germany
Donkey IgG anti-rat IgG (H+L)-Cy3	715-165-151	Dianova, Hamburg, Germany

Table S1: Antibodies used in the study.
